# Measuring Quality of Life Using Patient-Reported Outcomes in Real-World Metastatic Breast Cancer Patients: The Need for a Standardized Approach

**DOI:** 10.3390/cancers13102308

**Published:** 2021-05-12

**Authors:** Marloes E. Clarijs, Jacob Thurell, Friedrich Kühn, Carin A. Uyl-de Groot, Elham Hedayati, Maria M. Karsten, Agnes Jager, Linetta B. Koppert

**Affiliations:** 1 Academic Breast Cancer Center, Department of Surgical Oncology, Erasmus MC Cancer Institute, 3015 GD Rotterdam, The Netherlands; m.clarijs@erasmusmc.nl; 2 Department of Oncology-Pathology, Karolinska Institute and Karolinska University Hospital, 171 76 Stockholm, Sweden; jacob.thurell@ki.se (J.T.); elham.hedayati@ki.se (E.H.); 3 Department of Gynecology with Breast Center, Charité—Universitätsmedizin Berlin, Corporate Member of Freie Universität Berlin and Humboldt-Universität zu Berlin, 10117 Berlin, Germany; friedrich.kuehn@charite.de (F.K.); maria-margarete.karsten@charite.de (M.M.K.); 4 Department of Health Technology Assessment, Erasmus School of Health Policy & Management, Erasmus University Rotterdam, 3062 PA Rotterdam, The Netherlands; uyl@eshpm.eur.nl; 5 Academic Breast Cancer Center, Department of Medical Oncology, Erasmus MC Cancer Institute, 3015 GD Rotterdam, The Netherlands; a.jager@erasmusmc.nl

**Keywords:** metastatic breast cancer, quality of life, patient reported outcomes

## Abstract

**Simple Summary:**

Metastatic breast cancer (MBC) remains incurable despite treatment improvements. The health-related quality of life is a multidimensional entity which covers physical, psychological and social dimensions. It is an important outcome particularly in patients with metastatic disease, as the primary goal of therapy is no longer curation, but to provide the best possible quality of life weighted against treatment risks and adverse symptoms. Patient-reported outcomes reflecting the quality of life are usually measured with validated questionnaires to evaluate treatment strategies based on symptom burden and to improve care delivery. This review shares insights into the role of patient-reported outcome measurements in MBC patients and describes the heterogeneity of current questionnaires. We conclude that an up-to-date and standardized outcome set is needed, containing relevant domains referring to individual needs to improve the quality of life assessment among MBC patients. This is a prerequisite to learn about how they could impact the clinical care pathway.

**Abstract:**

Metastatic breast cancer (MBC) patients are almost always treated to minimize the symptom burden, and to prolong life without a curative intent. Although the prognosis of MBC patients has improved in recent years, the median survival after diagnosis is still only 3 years. Therefore, the health-related quality of life (HRQoL) should play a leading role in making treatment decisions. Heterogeneity in questionnaires used to evaluate the HRQoL in MBC patients complicates the interpretability and comparability of patient-reported outcomes (PROs) globally. In this review, we aimed to provide an overview of PRO instruments used in real-world MBC patients and to discuss important issues in measuring HRQoL. Routinely collecting symptom information using PROs could enhance treatment evaluation and shared decision-making. Standardizing these measures might help to improve the implementation of PROs, and facilitates collecting and sharing data to establish valid comparisons in research. This is a prerequisite to learn about how they could impact the clinical care pathway. In addition, the prognostic value of intensified PRO collection throughout therapy on survival and disease progression is promising. Future perspectives in the field of PROs and MBC are described.

## 1. Introduction

Breast cancer is the most common cancer diagnosis among women, with a yearly incidence rate of 47.8 per 100,000 females worldwide that is still gradually increasing [1]. The past few years have seen rapid improvements in treatment strategies for breast cancer, both in the area of locoregional and systemic treatment. Although survival rates of early-stage breast cancer have increased over the last few years [2,3], there remains a group of patients with incurable disease. Globally, metastatic breast cancer (MBC) comprises 5–10% of breast cancer patients at the time of diagnosis, and 20% to 50% of primary breast cancer patients will eventually develop metastatic disease [4,5]. Unsurprisingly, metastases are the worldwide major cause of death in breast cancer patients with a mortality rate of 13.6, resulting in more than half a million deaths in 2020 [6]. The estimated 5-year overall survival in MBC is 27%, which is still particularly poor [7]. However, therapeutic advances have also resulted in better outcomes for MBC patients, such as modest survival improvements, although without a curative intent [5,8,9]. Goals of therapy include diminishing symptoms, delay of disease progression, and prolongation of overall survival with the least negative impact on quality of life as possible [4]. Breast cancer patients face difficult challenges throughout their trajectory of disease, including numerous physical symptoms, emotional distress and impaired daily functioning [8,9]. These physical and psychosocial consequences of breast cancer diagnosis and treatment are reflecting the health-related quality of life (HRQoL), which has been increasingly recognized as an important endpoint in cancer treatment [10,11]. In MBC patients, the disease itself causes quality of life limiting symptoms and together with treatment-related symptoms, the impact on HRQoL may even be more substantial. The recently published Decade Rapport of Cardoso and colleagues showed a decline in overall quality of life in MBC patients over the last decade, based on a quantitative analysis of the EuroQol questionnaire [6]. The authors believe that this is as a result of unmet needs, less support and inconsistency of reported HRQoL data in MBC patients. The HRQoL is typically evaluated by patient-reported outcomes (PRO) and can be assessed by using validated instruments known as patient-reported outcome measurements (PROMs). The use of PROs has been associated with better patient satisfaction, quality of care and health outcomes [12]. While general cancer-related PRO measures have been used in MBC research, often to compare novel treatment strategies, previous studies recommend standardized and disease-specific HRQoL assessment methods [13,14,15]. In MBC patients with a future perspective of living longer with metastatic disease in particular, signaling changes in HRQoL during treatment is of great importance to maintain the quality of life weighed against the treatment benefits and toxicity. This demands a different approach compared to early stage breast cancer patients and emphasizes the need for an up-to-date HRQoL instrument dictated to patients with MBC.

The goal of this review is to describe the current use of questionnaires in real-world MBC patients by providing an overview of the available literature. We will highlight the importance of routinely monitoring appropriate PROs throughout treatment, including the implications and benefits of using PROs in daily clinical practice. This review concludes with opportunities and recommendations for the harmonized approach of HRQoL measurement applicability in MBC patients in clinical breast cancer care.

## 2. Materials and Methods

### 2.1. Literature Search Strategy

This paper is partly based on a systematic literature search using different online resources; MedLine, Web of Science Core Collection, Cochrane database and Embase. Search terms used were ‘metastatic breast cancer’ AND ‘quality of life’ AND ‘questionnaire’ OR ‘patient reported outcome’ OR ‘quality of life assessment’, and additional related terms to maximize the sensitivity of the search. The search was conducted in December 2020 and a total of 1736 articles matched the search term.

### 2.2. Patient-Reported Outcomes

Patient-centered health care is the cornerstone of current cancer care, and underlines the importance of collecting PROs [13]. PROs are defined as direct feedback on a patient’s health condition from a patient’s perspective and, therefore, PRO scores reflect the individual HRQoL without external interpretation [16]. Since the entrance of the HRQoL concept, several research organizations have developed questionnaires to transform this subjective concept into measurable scores. Some questionnaires can be used additionally for specific purposes, for example the EuroQoL Five-Dimension Scale (EQ-5D) to calculate health-utility scores for cost-effectiveness analyses or the BREAST-Q^®^ to evaluate breast surgery [17,18]. Besides questionnaires that are applicable for various diseases, more condition-specific instruments have been developed including breast cancer. An overview of available and validated questionnaires used in cancer patients is shown in Table 1.

Each instrument has specific questions attributed to domains that cover health issues, for example physical symptoms, daily functioning or emotional wellbeing. Thus, answers to every specific domain result in an individual score. Scores of separate domains can be summarized to generate a total score, and in general higher scores reflect better HRQoL. PROs were primarily invented to evaluate treatment strategies and thereby support clinical decisions. The collection of PROs at standard time points provides short-term information on treatment and disease burden, but longitudinal collection can also signal changes over time, which is helpful in starting a conversation at the outpatient clinic about certain domains wherein distress is identified [25]. With the growing experience in PROs over the last few years, they have earned their place as an important outcome in cancer research [10,11]. Many institutes worldwide have already integrated routine PRO monitoring with standardized outcome sets into patient portals and electronic systems, following an initiative of The International Consortium for Health Outcomes Measurement (ICHOM) [26]. ICHOM developed standardized outcome sets for a range of diseases, not necessarily with the intention to devise new outcomes measures, but to align on which well-validated PROMs providers and clinicians should use. A standard set for primary breast cancer already exists, and although a metastatic set has been composed for other cancers, this has not yet been done for MBC [26].

### 2.3. Inclusion and Exclusion Criteria

The criteria for inclusion in this review were studies published in the last 20 years and using questionnaires or patient-reported outcomes to evaluate quality of life in real-world MBC patients. The value of PROs as outcomes in clinical trials comparing systemic treatment regimens or other interventions in MBC patients have already been described in previously published reviews [27,28,29]. Although clinical trials evaluating PROs provide important insights in quality of life, the use of PROMs in such studies may serve a different purpose than the use for monitoring during daily clinical care. It was decided to focus on real-world MBC patients and that this specific topic was beyond the scope of the review. Articles based on clinical trials were, therefore, excluded. Studies focusing on locally advanced breast cancer only or focusing on other cancer types were not selected. Advanced breast cancer refers to both MBC (distant dissemination of the disease) and locally advanced disease. Locally advanced breast cancer includes primary cancers with extensive nodal or skin involvement, and is in general treated with a curative intent. Even though there is a risk of recurrent disease with distant metastasis in the following years [4], the main subject of this current paper is only MBC. Studies that included both non-metastatic and metastatic patients, but analyzed them separately, were also included.

## 3. Results

### 3.1. Included Articles

**Figure 1:**
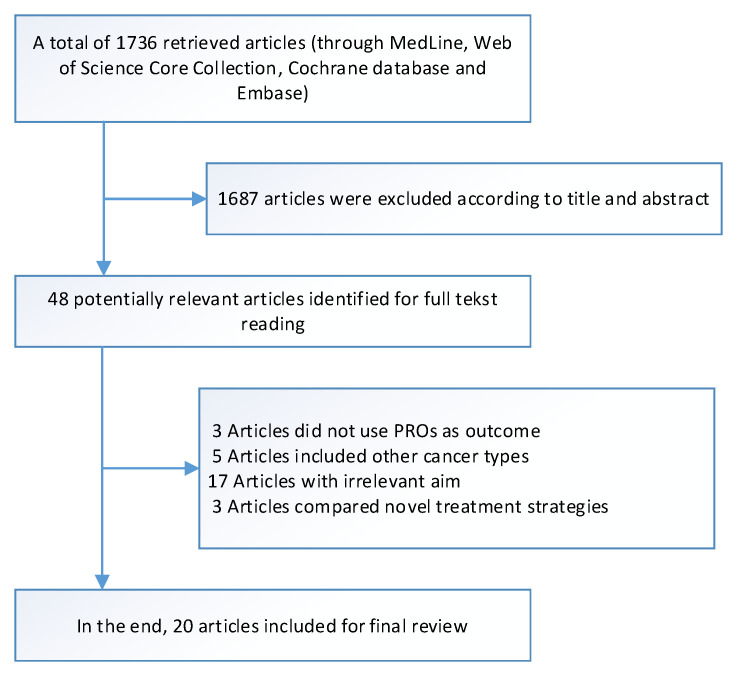
Flow diagram of relevant article selection.

Selection based on title and abstract was done by two researchers (M.C. and L.K.). In total, 48 articles were accepted for full text reading by both authors of which twenty articles ultimately met the inclusion criteria and are discussed throughout this review, see Figure 1.

### 3.2. Study Characteristics

The results of the literature search for citations meeting the inclusion criteria are summarized in Table 2. Six articles used PROs for evaluating the HRQoL during treatment, not necessarily to compare different treatment strategies, but to observe the influence of treatment on the QoL in MBC patients. Fourteen of the selected articles presented cross-sectional questionnaire-based studies.

### 3.3. Questionnaires and Patient-Reported Outcome (PRO) Domains

The European Organisation for Research and Treatment of Cancer Quality-of-Life Questionnaire (EORTC QLQ-C30) was most frequently used in studies [30,31,32,33,50], often supplemented by the EORTC QLQ-BR23 [34,35,36,37]. Other questionnaires that were identified, often combined with others, included the short form (SF-36) [31,38], Functional Assessment of Cancer Therapy–Breast Subscale (FACT-B) [39,40,41,42] and EuroQoL (EQ)-5D [39,45,46]. Several articles used other individual and less common PRO questionnaires. One study investigated the differences between HRQoL scores of 68 MBC patients compared to the general population, using multiple validated questionnaires. Of 96 included patients, 68 patients were diagnosed with MBC and 31 were receiving a form of adjuvant therapy. Analyses were done for the complete cohort as HRQoL did not differ between the MBC and adjuvant therapy group. Lower HRQoL scores were found in MBC patients across all used instruments and the EORTC QLQ-C30 captured most aspects of HRQoL. However, no adjustments for possible confounders, such as type of therapy or comorbidities were made in their univariate analysis [37]. Age above 65 and one or more comorbidities were associated with lower EQ-5D scores according to the stratified analysis of Claessens et al., but their sample size was not sufficient for multivariate regression analyses. The latter could probably have better explored the relationship between potential risk factors and HRQoL [43]. Different results were found in two other studies, in which older women had better psychological symptom scores than younger women [41,47]. According to the study of Costa et al., pain was prevalent in early-stage, locally advanced and metastatic disease, but only correlated with a decrease in QoL among MBC patients in a separate analysis [35].

### 3.4. Impact of Treatment on Health-Related Quality of Life (HRQoL)

#### 3.4.1. Chemotherapy, Endocrine and Targeted Therapy

Six studies assessed the HRQoL in MBC patients undergoing systemic therapy, either to evaluate single therapy or to compare endocrine and chemotherapy. The studies were difficult to compare due to heterogenic study design and outcome measurements. Amado et al. concluded that patients with low performance status at baseline seemed to have the greatest benefit on HRQoL following oncological treatment [38]. Although measured with different PRO instruments, similar results were found in the study of Cleeland et al. They also found younger age to be associated with greater symptom severity and reduced HRQoL. After adjustment for age, no difference in PRO outcomes were observed between the chemotherapy and endocrine therapy groups [49]. Chemotherapy was associated with greater symptom severity and lower functional wellbeing than endocrine or targeted therapy in two studies [34,41]. The overall quality of life measured with the EORTC QLQ-C30 after treatment did not differ from before for all treatments [34]. Kokkonen et al. also found worse physical functioning in MBC patients receiving chemotherapy, and the most common symptoms included pain and fatigue. However, most patients also underwent breast surgery prior to systemic treatment and they did not adjust for additional endocrine or targeted therapy [31]. HRQoL scores were higher in patients receiving chemotherapy versus supportive care, but results must be interpreted with caution as 40% of the supportive care group received palliative radiotherapy which possibly decreased the HRQoL [36].

#### 3.4.2. Bisphosphonate Treatment and Bone Metastasis

Bone metastases frequently occur in MBC patients for which there are a variety of treatment options, including bisphosphonates. Three studies focused on MBC patients with bone metastasis and showed lower QoL compared to patients without bone metastasis [41,43,50]. One study indicated that bisphosphonate treatment was associated with better wellbeing. The authors did not adjust the results for additional treatments, disease progression and several other factors that could impact the association. Results were possibly biased as patients with only visceral metastasis were also included, although biphosphonate treatment is not effective in these cases [39]. The study of Barnadas et al., a subanalysis of patients receiving specific treatment for bone metastatis versus no treatment revealed clinically significant improvement in HRQoL [48].

### 3.5. Depression and Anxiety

The Hospital Anxiety and Depression Scale and Back Depression Inventory were mostly administered in screening for depression and anxiety, but also for general QoL outcomes [38,40,42,45,46,47]. Overall, mean HADS scores for anxiety were higher, signifying greater burden, than depression scores. Love et al. found similar outcomes for the HADS and BDI questionnaire, although the BDI-SF performed better in screening for depression in MBC patients [45]. Multiple regression analysis by Shin et al. showed a higher prevalence of clinical depressive and anxiety symptoms in patients receiving chemotherapy compared with endocrine therapy. However, chemotherapy was not an independent risk factor of these outcomes and lower QoL in the chemotherapy group possibly caused higher levels of depression and anxiety [42]. In patients living 5 years or longer with MBC, the overall quality of life was good, but low scores were found in emotional subscales. Half of the eligible patients did not respond due to illness severity and the authors did not distinguish between type of therapy [40].

### 3.6. Disease Progression

The retrospective study of Müller et al. showed that disease progression was associated with a more than 2-fold risk (hazard ratio of 2.22) of experiencing minimally important deterioration in HRQoL based on the EORTC QLQ-C30. Regarding mean HRQoL scores, no differences were found between patients with and without progression [33].

### 3.7. Hospitalization

The study of Lima et al. assessed the HRQoL in hospitalized MBC patients. The global health status averaged 32.04, which is lower than in other studies of MBC patients in the outpatient setting. The global health status was significantly lower in stage IV patients. This is probably explained by the fact that hospitalization itself and the symptoms causing admission reduce the HRQoL [32].

## 4. Discussion

As the breast-cancer specific PRO measurements were developed for early-stage breast cancer, translating them to patients with MBC can be challenging and difficult [51]. The 20 papers that were included, used 17 different PROMs to monitor the QoL in real-world MBC patients. As previously mentioned, clinical trials using PROs to evaluate novel or different chemotherapy regimens in MBC patients were excluded. However, the questionnaires used in real-world MBC patients as described in this current review are in line with PROs used in randomized clinical trials according to previously published systematic reviews [14,27,28,29,52]. These results together show that there is still large heterogeneity in instruments used for PRO measurement in MBC patients. The various questionnaires, subscales and scoring systems leading to disaggregated data of PROs may complicate the answers to research questions or hypotheses [12,53]. A standardized outcome set may be a prerequisite to improve the interpretation of PROs in daily clinical care but also in clinical trials and to recognize them as an important endpoint.

Most MBC patients do not qualify for surgical treatment and the effect of surgical removal of the primary tumor on survival and quality of life is not convincing [54,55,56]. Chemotherapy, endocrine therapy and targeted immunotherapy with biological agents have been modified over recent years with increasing effectiveness [57,58]. For oncologists administering novel treatments with respect to the benefit-risk ratio for patients is challenging. Because treatment options sometimes have similar efficacy based on traditional outcomes such as survival or tumor response, but have different toxicity, new indicators such as HRQoL or symptom burden can be used to support treatment decisions [59]. However, the impact of systemic agents on the HRQoL remains a subject of controversy. Some studies have shown that endocrine therapy and trastuzumab can improve the overall QoL following treatment by diminishing symptoms [34,60]. Chemotherapy is effective in relieving cancer-related symptoms and disease control, but can negatively impact well-being, especially in patients with a poor baseline HRQoL. A study of 378 women with advanced breast cancer receiving chemotherapy, showed that appetite and physical wellbeing at baseline were independent predictors of overall and progression free survival. Women with poor baseline HRQoL received fewer cycles of chemotherapy and experienced more toxicity. Thus, HRQoL itself can be an independent predictor of chemotherapy efficacy [36,61].

Despite promising effects, intense treatments are causing severe side effects which can also deteriorate the patient’s quality of life, sometimes making it worse enough to discontinue treatment. One study showed treatment non-adherence in one-third of breast cancer patients receiving endocrine therapy due to adverse events [62]. Therefore, it is important to include patients in the decision-making process and jointly decide whether they are eligible for more or less aggressive treatment regimens, and how they can trade-off side effects and QoL. QoL monitoring with use of PROs can be helpful in this process, as worsening of adverse symptoms can be signaled early and information as well as guidance of the patient might be improved [59]. Unfortunately, today’s available PRO instruments for MBC patients do not always seem sufficient to detect changes in HRQoL. Therefore, demonstrating possible variation in HRQoL scores between different systemic treatments in clinical trials is difficult and complicates clinical decision-making [52,63].

Some studies that examined the completion of questionnaires, however, found low rates of patient adherence, sometimes associated with disease severity and the inability to complete questionnaires [12]. Another possible cause is the lack of information given about the potential and importance of PROs as well as the meaning of the resulting scores. If outcomes are discussed or explained by physicians in the outpatient clinic, poor adherence to completion of questionnaires will most likely be avoided. Patients might feel uncomfortable starting a conversation about emotional or social problems, unless their physician initiated a discussion on these topics. HRQoL questionnaires may contribute to ease these conversations by being a helpful tool to identify problematic health issues [25]. To overcome the aforementioned barriers, creating dashboards that visually present the scores of a specific patient in simplified pictures, graphics or tables can be more informative than only abstractive numbers. Additionally, individual patient scores can be compared with scores of breast cancer patients with the same biological or treatment-related characteristics also known as reference scores [64]. Normative data reflect outcomes of a population unencumbered by a disease or specific condition, and can be used by both clinicians and patients to provide more context when interpreting PROs. The consensus in cancer research is still that conclusions are based on statistical significant differences. However, in the evaluation of PROs it is not only important to take the statistical significance into consideration, but also the extent to which these differences are clinically meaningful, also known as minimal clinical important differences (MCID). MCIDs are the smallest changes in PROM scores, that are important and relevant enough for an individual patient to justify a modification in patient management [65]. MCIDs can be determined for individual questionnaires and both MCIDs and thresholds can support the clinical evaluation of PROs. To date, these reference data for MBC patients are scarce.

## 5. Future Perspectives

In a constantly improving and changing health care system, where patient-centered care is being more prioritized, measuring HRQoL should be a routine clinical assessment. Although many researchers and clinicians agree, a wide variety of questionnaires resulting in different values make it difficult for healthcare professionals to become familiar with PRO data and could also hamper the exchange of information between treating physicians and disciplines. Particularly in the field of breast cancer research, with major developments in PROs over de last years, the necessity for comparable outcome data is apparent. Yet traditional questionnaires each result in an instrument-specific score that is difficult to compare. One approach to address this challenge is the development of common metrics for the specific outcomes of interest such as fatigue and depression [66,67]. Common metrics are statistical models based on modern test theory (item response theory, IRT), that cover multiple questionnaires and, therefore, allow different questionnaires to be scored on a common scale. However, standardizing outcome sets has also been proven effective and efficient, enabling the comparison of quality of life scores between institutes [13,26].

Some clinicians are concerned about the patient burden due to frequent questionnaire assessments. However, it is likely that intensified surveillance and detection of short-term changes in metastatic disease will require shorter time intervals and more frequent surveys than in early-stage breast cancer patients. For example, in a landmark study evaluating an intensified digital PRO elicitation, Denis et al. defined a time interval of only one week between self-reports during lung cancer treatment [68]. Initiatives such as the EORTC or PROMIS developed IRT-based, construct-specific measurement models and established standardized item banks, offering the prospect of a less burdensome and more valid PRO assessment through tailored short forms and computerized adaptive testing (CAT) [69,70]. CAT describes an assessment of the respective construct (e.g., pain, fatigue, physical functioning) which specifically asks questions deemed most informative based on currently available information. This results in greater precision without extending the test length which might decrease the effort, respectively, the burden, for patients to answer the questionnaires [71]. Even if institutions struggle to implement CAT due to technical prerequisites, an IRT-based standard set for metastatic breast cancer would only need to include the domains of interest rather than specific questionnaires or items. Use of common metrics and construct-specific item-banks would enable comparable data despite different items or instruments.

Another interesting topic is the additional prognostic value of PRO-supported care on survival. Studies have shown an increase in overall survival through intensified HRQoL monitoring using, among others, the EQ-5D and FACT-L [68,72]. In a randomized controlled trial by Basch et al. including 766 metastatic cancer patients, digital PRO assessment for symptom monitoring in the intervention group led to an alert email to the treating center in case of symptom deterioration. The control group received the usual care with symptom monitoring during routine clinical visits only. Patients in the PRO group showed significantly higher HRQoL scores at 6 months after enrollment and overall survival increased by 5 months compared with usual care [72,73]. Moreover, in a study among 121 lung cancer patients taking a similar approach, Denis et al. presented an overall survival of 22.5 months in the PRO group compared to 14.9 months in the control group [68]. Against this backdrop, one can assume that PRO monitoring might facilitate an early detection of symptoms associated with adverse events or disease progression, thus enabling timely countermeasures, which ultimately result in improved overall survival. In addition, a recent meta-analysis of Efficace et al. identified several PRO domains (e.g., fatigue, appetite loss) to be independent predictors for overall survival in metastatic cancer patients [74]. The strongest association was found with physical functioning, showing a 12% increase in risk of death for every 10-point decrease on a scale of 1 to 100. The results underline the importance of baseline measurements and the systematic administration of PROs to also capture prognostic information. However, PROs comprise more than only symptom burden and, apart from possible aforementioned survival benefits, minimizing the physical and psychosocial impact of MBC is important in itself. Studies performed in MBC patients to identify the optimal patient-centered approach for electronic PRO collection in routine clinical care concluded that physical symptoms or treatment toxicity are not always a priority, but financial concerns or emotional well-being even so, and that PRO collection should be multidimensional [30,75]. The EORTC QoL questionnaires are used frequently in cancer research and the need for an MBC specific questionnaire has not gone unnoticed by the EORTC workgroup. They are currently working on a comprehensive questionnaire for HRQoL assessment in MBC patients, with the aim to conduct phases 1 to 3 of the module development process in the next two years. The European Innovative Medicines Initiatives Funded Health Outcomes Observatory (H2O) project is a recently developed initiative to improve the quality of care by creating ‘health outcomes observations’, which also aims to collect standardized health data, among which (metastasized) breast cancer.

## 6. Conclusions

Current treatment developments for MBC patients can impact the symptom burden and quality of life, emphasizing the importance of HRQoL measurements. We believe that an important step in accelerating value-based health care for MBC patients is to implement a standard set of PROs for routine clinical care. An appropriate and standardized set could be used in evaluating treatment strategies and making clinical decisions. It will increase the interpretability of PROs and allow for comparisons in MBC outcomes. Eventually, national and international benchmarking will help to develop a stronger theoretical foundation for future research and lead to improvements in daily breast cancer care. The value of PROs and specific domains for the prediction of survival outcomes or disease progression should be studied further.

## Figures and Tables

**Table 1:** Concrete examples of patient-reported outcome measurements (PROMs) used in cancer patients with questionnaire characteristics.

Questionnaire	Subscales	No. of Items	Response Scale (Likert-Scale)	Scoring System	Recall Period
EORTC QLQ-C30 [19]	Generic	30	4-point	0–100	Past 7 days to 4 weeks
EORTC QLQ-BR23 [20] (updated EORTC QLQ-BR45)	Breast Cancer Subscale	23	4-point	0–100	Past 7 days to 4 weeks
FACT-ES [21]	Endocrine Therapy Subscale	46	5-point	0–184	Past 7 days
FACT-B [22]	Breast Cancer Subscale Trial Outcome Index	37	5-point	0–148 0–96	Past 7 days
EQ-5D-5L EQ-5D-3L [17]	Generic	6	5-point 3-point	Health states and VAS-score 0–100	Today
BREAST-Q (pre- and post-operative) [18]	Mastectomy, Breast Conserving Therapy and Reconstruction module	4–11 (depending on subscale)	3, 4 and 5-point	0–100	Past 7 days
MOS SF-36 [23]	Generic	36	3, 5 and 6 point	0–100	Past 4 weeks
RSCL [24]	Generic	39	4 point	0–135	Past 7 days

EORTC-QLQ = European Organisation for Research and Treatment of Cancer Quality-of-Life Questionnaire C-30, Breast cancer-23 and Breast cancer-45; FACT-B/ES = Functional Assessment of Cancer Therapy–Breast and Endocrine Subscale; EQ-5D = EuroQoL-5 dimensions; MOS SF-36 = Medical Outcomes Study-Short Form 36; RSCL = Rotterdam Symptom Checklist.

**Table 2:** Instruments used for health-related quality of life (HRQoL) measurements in metastatic breast cancer (MBC) patients.

**Selected Article**	**Study Objective**	**Study Design**	**Study Population**	**Administered PROM**
Aranda, S. et al., 2005 [30]	To identify the support- and information needs in urban MBC patients	Cross-sectional multicenter study	105 Australian patients from four different hospitals in Melbourne with MBC. 61% response rate.	EORTC QLQ-C30 and SCNC
Kokkonen, K. et al., 2017 [31]	To assess the functional capacity and quality of life of Finnish MBC patients	Cross-sectional observational study	128 Finnish patients with ongoing treatment for MBC, treated at Helsinki University Hospital. 61% response rate.	BDI, HAQ, RAND SF-36 and EORTC QLQ-C30
Lima, E.O.L. et al., 2020 [32]	To assess QoL in hospitalized MBC patients	Cross-sectional observational study	199 (145 with stage IV) Brazilian patients with locally advanced (stage IIB, IIIA, B and C) or MBC (stage IV) that were hospitalized in Rio de Janeiro.	EORTC QLQ-C30
Müller, V. et al., 2018 [33]	To assess the impact of disease-progression on HrQoL in MBC patients	Retrospective, longitudinal, observational study	326 MBC patients from the PRAEGNANT database	EORTC QLQ-C30
Adamowicz et al., 2020 [34]	To assess QoL in MBC patients dependent on treatment-choice	Prospective, multicenter observational study	351 Polish MBC patients undergoing first-line palliative chemotherapy, HER2-treatment or endocrine therapy at two hospitals in Gdansk.	EORTC QLQ-C30 and BR23
Costa, W.A. et al., 2017 [35]	To assess the influence of pain on QoL in breast cancer patients undergoing treatment	Cross-sectional study	400 Brazilian breast cancer patients from one hospital were included. Of these, 160 patients had MBC and were analyzed separately.	McGill Pain Questionnaire, EORTC QLQ-C30 and BR-23
Karamouzis, M. et al., 2007 [36]	To evaluate QoL parameters in patients with MBC	Prospective, randomized, single-center study	210 women with MBC patients receiving chemotherapy vs supportive care	EORTC QLQ-C30 and BR23
Wallwiener, M. 2016 et al. [37]	To assess the HRQoL of MBC patients and breast cancer patients under adjuvant treatment compared with the general population.	Cross-sectional, single-center study	96 German patients with MBC or under adjuvant treatment for breast cancer. Response rate 80%.	EORTC QLQ-C30, and BR23, EQ-5D-5L and EQ-VAS.
Amado, F. et al., 2006 [38]	To evaluate changes in QoL among MBC patients receiving treatment	Prospective, observational survey study	40 Brazilian MBC patients that were about to start palliative treatment. Data was collected before start (baseline) and after 6 and 12 weeks of treatment.	BDI, SF-36
Ecclestone, C. et al., 2016 [39]	To examine the symptom burden and QoL in MBC patients	Cross-sectional observational study	174 Canadian MBC patients with only bone metastasis compared to MBC patients with visceral and/or bone metastasis.	ESAS, FACT-B
Meisel, J.L. et al., 2012 [40]	To evaluate psychological adjustment of women living long-term with metastatic disease	Cross-sectional study	28 eligible US women, of which 18 completed the questionnaires.	HADS, IES-R, DUFSS, FACT-B
Reed, E. et al., 2012 [41]	To explore QoL, experience of care and support needs in MBC patients	Cross-sectional study	235 women with MBC off two U.K. cancer centers (N = 110) and online survey (N = 125).	FACT-B
Shin, J.A. et al., 2016 [42].	To study the QoL, depression and anxiety in patients with MBC	Cross-sectional, study	140 US MBC patients, stratified by endocrine therapy (40) and chemotherapy (100)	HADS, FACT-B (TOI)
Claessens, A.K. et al., 2020 [43]	To evaluate the QoL using the EQ-5D-3L in Dutch advanced breast cancer patients	Cross-sectional study	92 Dutch patients with MBC were analyzed.	EQ-5D-3L
Slovacek, L. et al., 2010 [44]	To evaluate global QoL and depression among MBC patients	Prospective, cross-sectional study	41 Czech patients in a program of palliative cancer care	EQ-5D, ZSDS
Love, A.W. et al., 2004 [45]	To identify possible depression in MBC patients	Cross-sectional screening study	74 patients with depression were identified.	HADS, BDI-SF
Park, E.M. et al., 2018 [46].	To determine factors associated with anxiety and depression in young MBC patients	Cross-sectional study	54 women with de novo MBC from an ongoing prospective, multicenter cohort of women diagnosed <40 years.	HADS, CARES-SF, MOS
Turner, J. et al., 2005 [47]	To investigate psychosocial aspects of MBC	Cross-sectional study	66 women diagnosed with MBC under ongoing treatment at two large hospitals in Australia.	HADS, IES, CARES-SF, MSAS
Barnadas, A. et al., 2019 [48]	The applicability of the BOMET-QoL-10 measure in MBC patients	Prospective, observational, multicenter study	172 breast cancer patients with bone metastasis at 15 GEICAM hospitals in Spain.	BOMET-QoL-10
Cleeland, C.S. et al., 2014 [49]	To evaluate baseline PROs in patients with MBC and first-line hormonal, targeted or chemotherapy	Cross-sectional study	152 patients of VIRGO observational study, 104 received chemotherapy and 48 endocrine therapy.	MDASI, WPAI:SHP, RSCL

BDI = Beck Depression Inventory; SCNC = Supportive Care Needs Survey; WPAI:SHP = Work Productivity and Activity Impairment Questionnaire; HAQ = Health assessment questionnaire; ESAS = Edmonton Symptom Assessment System; MDASI = MD Anderson Symptom Inventory; IES-R = Revised Impact of Events Scale; DUFSS = Duke-University of North Carolina (UNC) Functional Social Support; ZSDS = Zung self-rating depression score; MSAS = Memorial Symptom Assessment Scale; CARES-SF = Cancer Rehabilitation Evaluation System-Short Form; MOS = Medical Outcomes Study Social Support Survey; BOMET-QoL-10 = Bone Metastasis Quality of Life measure; HADS = Hospital Anxiety and Depression Scale; GEICAM = Spanish Breast Cancer Research Group.

## Data Availability

Not applicable.
